# The balancing act of urban conservation

**DOI:** 10.1038/s41467-020-17539-0

**Published:** 2020-07-29

**Authors:** Katherine J. Turo, Mary M. Gardiner

**Affiliations:** The Ohio State University, Department of Entomology, 2021 Coffey Road, Columbus, OH 43210 USA

**Keywords:** Conservation biology, Urban ecology

## Abstract

As investment in urban conservation grows, researchers must balance the needs of residents and conservation targets. We discuss some of the challenges we have encountered and the importance of taking a transdisciplinary approach informed by design and social knowledge.

Urban greenspaces are increasingly considered as conservation habitats. In particular, vacant lots offer valuable opportunities (Fig. 1a). Vacant lots can be transformed into urban farms, rain gardens, and “pocket prairies” (Fig. 1b) in order to conserve biodiversity, deliver ecosystem services, and improve the equitable distribution of high-quality living conditions amongst city residents^1–5^. Urban conservation models champion an integrated social and ecological approach in creating greenspaces to account for the combination of biophysical, socioeconomic, and cultural factors^6–8^ that shape city ecosystems. However, balancing socioecological theory and praxis is difficult^4,9^, and strategies for implementation are not universally applicable.

**Fig. 1:**
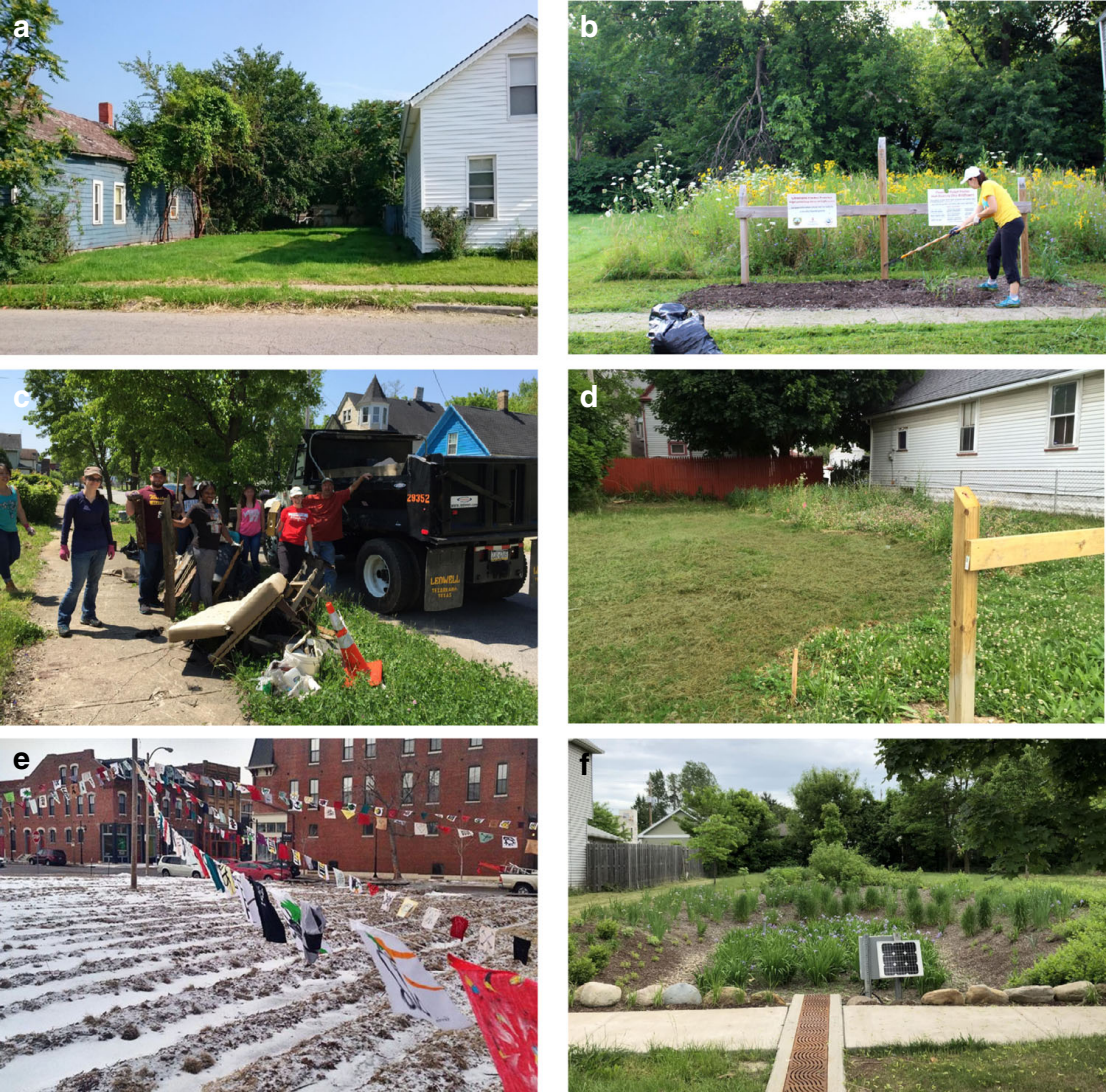
Urban greening projects should anticipate challenges and plan to work collaboratively with stakeholders to address them. Post-industrial cities such as Cleveland, Ohio, USA face the significant challenge of managing thousands of vacant lots (i.e. parcels where pre-existing structures have been torn down and replaced by minimally-managed vegetation) (**a**). Although typically viewed as blight, these sites do offer opportunities to conserve urban biodiversity. In 2014, the Gardiner Laboratory established 64 conservation habits on vacant land across the city of Cleveland, including 32 sites seeded with native perennial wildflowers (**b**). Our cost to establish the 32 pocket prairies was approximately $2500 per site and included soil preparation, seeding, invasive plant management, and installing ‘cues to care’^11^ such as fencing and mulching. Maintaining cues to care required substantial time and financial investment. Trash, furniture, appliances, and other refuse are frequently dumped into urban lots and must be removed (**c**). Vandalism to signs, fencing, and vegetation is also common, especially in the springtime when native plantings have not bloomed yet and sites can appear weedy and unkempt (**d**). Similar aesthetic concerns can happen during the winter. Thus, it is imperative to work with local stakeholders to identify what design modifications can indicate year-round investment into a conservation site. For instance, greenspace managers at the Sunflower+Project: STL, in St. Louis, Missouri, partnered with local elementary schools to paint sustainability flags which hung above their over-wintering sunflower fields (**e**). However, even with substantial investment, vacant lot ecosystem management can remain a controversial endeavor. Although many residents view rain gardens established though a multi-million USD investment by the Northeast Ohio Regional Sewer District as valuable storm water management, others see a poor use of funds that raises health and safety concerns^4^ (**f**). Thus, we must work with urban residents and municipal governance as community developers to meet the needs of the diverse human ecosystem. Photograph E courtesy Richard Reilly.

Our insights are informed by a decade studying the ecology of vacant land within Cleveland, Ohio, USA, a post-industrial city that currently encompasses >27,000 vacant lots. In 2012, we received funding to evaluate eight economically-feasible strategies to manage vacant land, with the broad goals of improving habitat quality for arthropods, supporting ecosystem services^3^, and beautifying the city (Fig. 1). Despite familiarity with recognized socioecological frameworks, we struggled to apply recommendations to our project, particularly when establishing and maintaining native plants. We share our experiences of practical realities ecologists can face when attempting to follow best practices, and note the strategies employed by our team and others when implementing community-driven conservation (Fig. 2).

**Fig. 2:**
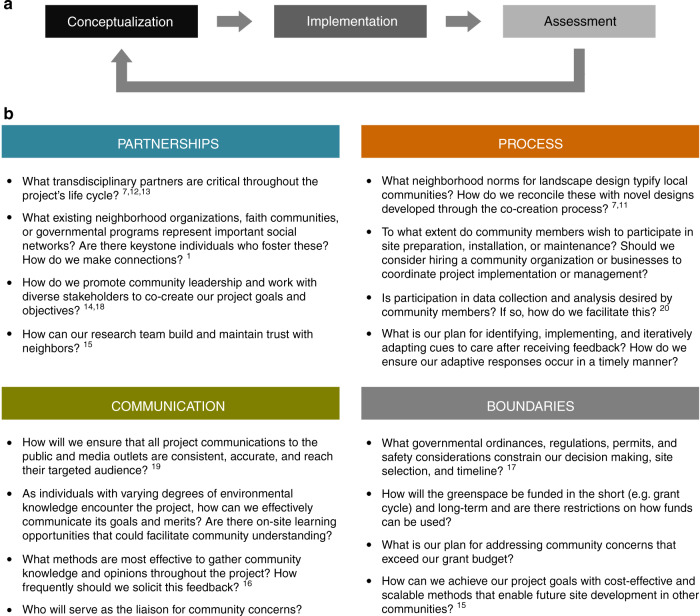
Guiding questions for ecologists planning future urban greening projects. A “for the city” paradigm for ecological research approaches urban conservation as an iterative community development process (**a**) for the benefit of urban residents and taxa of interest. Applying such frameworks can be difficult for scientists with disciplinary knowledge but little expertise working with a diverse set of stakeholders. We recognize that praxis often falls short of aspirational theory and provide the following set of questions and references (**b**) for consideration by practitioners embarking on new projects^1,7,11–20^. As urban conservation is complicated and context dependent, this is not an exhaustive list and references often apply to multiple questions. Moreover, we emphasize that both community development and urban conservation are long-term endeavors, not activities bounded by a research grant, and that iterative adaptions are critical to achieving positive outcomes.

## Co-creating habitat goals sets a project up for success

Paradigm shifts in urban ecology have emphasized the ethical responsibility of scientists to prioritize the city and its residents^8^ by investing in community development and investigating questions relevant to human interests. Therefore, we collaborated with government officials at the Cleveland Land Bank, city council members, non-profit organizations, city planners, and other community leaders over a 12-month period to identify potential sites for our urban conservation project and finalize habitat plans. Following this, we canvased one city block surrounding each of the 64 vacant lots included in our study to discuss our research plans with residents and replace sites that drew irresolvable concerns.

Despite endorsing a “for the city” paradigm^16^ (Fig. 2), our project was not universally well received by residents and our habitat plantings experienced vandalism, dumping, and public criticism (Fig. 1c, d). In hindsight, we realize that we expected residents to tolerate our newly grant-funded project when we should have co-created project objectives with residents. Residents’ goals for urban greenspaces may vary with their demographics, neighborhood cultural norms, and environmental knowledge^10,11^, and these goals may not automatically align with plans developed from an ecological perspective.

In order to respect this diversity, ecologists must proactively develop transdisciplinary partnerships^7,12,13^ with sociologists, landscape architects, urban planners, and economists. With these partners, projects can connect with community members through iterative listening sessions, neighborhood surveys, focus groups, and planning meetings that focus on community-building and long-term conservation gains^1,7,14^. While we sought partnerships during project development, we found that the scale of our study was too large for our team to effectively engage at the community level. If we had worked in fewer neighborhoods and prioritized connecting with neighborhood-scale organizations instead of city-wide governance, we would have better understood the concerns of neighbors. Likewise, we would have benefited from sociological expertise when assessing and responding to community feedback, instead of only when identifying best practices for our proposal. Furthermore, our focus on economic feasibility and large-scale implementation necessitated a simplistic habitat design with minimal management costs, but this proved unrealistic. Working closer with landscape architects would have helped us better anticipate and resolve neighbors’ aesthetic and safety concerns in our initial habitat design rather than through iterative modifications.

Urban conservation projects that effectively use neighborhood-scale organizational partnerships and invite community members to participate in goal setting and design are better primed for success. For example, the Burnham Wildlife Corridor in Chicago, Illinois partnered with the Field Museum and the Chicago Park District and synthesized 20 years of participatory action research on community perspectives to create their “Roots and Routes” initiative^1^. This program successfully aligned their conservation goals with residents’ perspectives on greening to simultaneously develop migratory bird habitat, gathering spaces for communities, and opportunities for youth engagement and employment^1^. In part, we attribute “Roots and Routes” success to their design-competition approach that allowed community groups to determine their own project goals and habitat plans; this should inspire future projects to creatively incorporate community perspectives during grant development.

## Project sustainability relies on community relationships

Co-creating a greenspace is only the beginning; conservation practitioners must also communicate progress and maintain community member’s trust throughout a project’s duration^15^. Despite residents’ involvement in the co-design process, feelings of “bait and switch” can arise if a developing habitat aesthetically diverges from their expectations. For instance, non-native weedy vegetation may become more abundant within a planting than anticipated. Thus, frequent discussions of all possible or transitory site outcomes, including visual representations of a habitat’s vegetation^9,14^, can help avoid feelings of contention or disinvestment. Likewise, research tools can be misunderstood and cause concern if not effectively described. For example, neighbors have expressed apprehensions that our native bee traps were releasing stinging insects when in fact they removed the insects for further study. This confusion could have been avoided by better communication at the project’s onset and throughout continued interactions with residents.

To effectively engage a large and diverse urban community, researchers must evaluate multiple options to share their activities and findings^14,16^. We created a project website, educational video, and social media presence; these have been successful in communicating with other researchers and the media but have largely failed to reach residents. We found that one-on-one discussions of project aims, progress, and outcomes through daily interactions on-site or at community events were far more effective, but still did not reach all stakeholders. For example, our research activity occurred during the day, limiting interactions with those who were away at work, and a high turnover rate in housing occupancy created an influx of new residents unfamiliar with the project. Also, residents may be more comfortable interacting with neighbors or local organizations rather than visiting researchers. To address these issues, scientists should consider pre-existing channels of communication (e.g. neighborhood watch groups, religious organizations, community centers) that are self-identified by community leaders.

Building community relationships requires more than disseminating information; researchers need to actively solicit community opinions in order to gauge and address needs. Regular polling of community opinions through focus groups or surveys can inform habitat management to help resolve community concerns such as aesthetics or perceived safety. While an ecologist may see diverse native plants flourishing, dense and tall plantings can inspire fear of criminal activity^17^ and community members may consider such habitats as eye-sores indistinguishable from abandoned properties^11^. Such issues can be mitigated through “cues to care”—the physical signs of intention and upkeep^11^ advocated by design professionals. These cues can indicate a greenspace has purpose, help combat negative perceptions, and illustrate community consideration^11^. Common practices such as signage, neatly-mown borders, fences, and/or mulched flower beds around a conservation site can convey a site’s purpose and contribute to community approval^9,11^. For example, abundant signage and neatly-mown edges were noted as significant factors promoting public support for urban meadows in Bedford and Luton, UK^9^. We employed similar cues to care and framed our pocket prairie habitats with a mown border, fence, and mulched roadside edge (Fig. 1b). Yet, we received complaints from residents who did not perceive our mown borders as intentional and assumed we had abandoned our mowing efforts. Concern was also expressed that the mulch was a health hazard as stray cats might use it as litter. This illustrates how widely recommended cues to care are not effective in all settings and failure to engage residents in management planning may result in confusion or elicit unanticipated, negative feedback. Conversely, if residents are involved in determining cues to care, creative solutions generating greater satisfaction can be found. For example, hand-painted flags designed by elementary school students were an effective cue to care for the off-season within a sunflower planting in St. Louis, Missouri^5^ (Fig. 1f).

It is important for community leaders, scientists, and neighbors to recognize the difficulty in reconciling a community’s diverging opinions of greenspace goals. Even with open communication, projects will face challenges in meeting community expectations. For instance, some Cleveland, OH residents prefer the tidy appearance of fabric flowers over the living vegetation of a habitat planting. After 4 years, we are still trying to develop a strategy to meet this concern. Meanwhile, we have also received many positive comments, with residents enjoying the color of our plantings, asking to pick flowers for bouquets, or declaring their general support for helping declining bees. We highlight these variable responses as both precaution and encouragement. It is unlikely that urban conservation sites will garner universal public support^2^, but iterative assessments and modifications of a site’s management or design can ameliorate some community concerns and shift how greenspace is viewed and valued long-term.

## Final remarks

Balancing the diverse needs of human and ecological systems is complicated, context-dependent, and relies upon partnership, detailed planning, and continued community engagement. Such an approach is critical to avoid driving a wedge between academics and urban residents, who may perceive researchers as outsiders charging in to “save” or “experiment on” inner-city neighborhoods. Although we are still learning how best to approach urban conservation, we recognize that connecting and co-creating with a broad group of stakeholders is a critical first step. Likewise, open lines of communication and timely management adjustments can help an urban greening project succeed. It is our hope that by approaching urban conservation as a community development process, we can collectively be better equipped to serve people and our taxa of interest.
